# Factors related to implementation of an interprofessional communication concept in thoracic oncology: a mixed-methods study

**DOI:** 10.1186/s12904-022-00977-6

**Published:** 2022-05-26

**Authors:** Katja Krug, Jasmin Bossert, Sophia Möllinger, Nicole Deis, Laura Unsöld, Anja Siegle, Matthias Villalobos, Laura Hagelskamp, Corinna Jung, Michael Thomas, Michel Wensing

**Affiliations:** 1 Department of General Practice and Health Services Research, Heidelberg University Hospital, Im Neuenheimer Feld 130.3, 69120 Heidelberg, Germany; 2 Department of Thoracic Oncology and Translational Lung Research Center Heidelberg (TLRC-H), Member of the German Center for Lung Research (DZL), Heidelberg University Hospital, Röntgenstraße 1, 69126 Heidelberg, Germany; 3 Medical School Berlin, Calandrellistr. 1-9, 12247 Berlin, Germany

**Keywords:** Interprofessional collaboration, Attitude, Communication, Advanced cancer, Implementation

## Abstract

**Background:**

An innovative patient-centred interprofessional communication concept with advanced lung cancer patients (Heidelberg Milestone Communication Approach, MCA) has been developed and implemented. Role changes and interprofessional communication are challenging in a busy outpatient oncology service. The aim of the study was to present attitudes to interprofessional collaboration of professions in thoracic oncology during the implementation of MCA and to explore factors and experiences healthcare team members associate with its implementation.

**Methods:**

In a longitudinal study, 3 of the 4 subscales of the validated German translation of the University of the West of England Interprofessional Questionnaire (UWE-IP-D) were collected prior to implementation of MCA (t0) with follow-up data collections at 4 months (t1), 10 months (t2) and 17 months (t3). Descriptive analysis included calculating subscale sum scores and categorizing each subscale into positive, neutral and negative attitudes. Interviews and focus groups on implementation and interprofessional collaboration in the context of MCA were conducted with healthcare staff. The topics were analysed deductively, guided by the Professional Interactions factor of the Tailored Implementation for Chronic Diseases (TICD) framework.

**Results:**

The survey with 87 staff (44 nurses, 13 physicians, 12 psycho-social staff, 7 therapists, and 11 others) participating at least once found heterogeneous attitudes. ‘Communication and Teamwork’ and ‘Interprofessional Relationships’ were characterized by primarily positive attitudes. Neutral attitudes to ‘Interprofessional Interaction’ were indicated by the majority of respondents. There were no differences between collection times. Fifteen staff members participated in the interviews and focus groups. The main interprofessional interaction factors associated with implementation concerned the knowledge of the MCA and the impact of the intervention on team roles, on information sharing and on transfer processes between wards. Adaptive processes led to a shift in the perception of responsibilities and interprofessional collaboration.

**Conclusions:**

Positive experiences and potential shortfalls in the implementation were observed. Future introductions of interprofessional communication concepts require further activities which should address the attitudes of healthcare professionals towards interprofessional care.

**Trial registration:**

DRKS00013469 / Date of registration: 22/12/2017.

**Supplementary Information:**

The online version contains supplementary material available at 10.1186/s12904-022-00977-6.

## Background

In spite of improved diagnostic and treatment options, lung cancer is associated with a limited prognosis as the diagnosis is often made at an advanced stage [1]. In light of this limited prognosis, cancer care poses major challenges for patients and informal caregivers as well as for healthcare staff due to the need to organise and prioritise different stages during the course of the disease. Effective interprofessional collaboration may help to optimise cancer care overtime. Interprofessional collaboration may contribute to patient-centred care and help to enhance end-of-life outcomes, such as quality of life and the amelioration of symptoms [2]. Other than benefits for patients, healthcare professionals (physicians, nurses, therapists) may also benefit from successful interprofessional collaboration through improved working climate and better workflow. Studies indicate that interprofessional collaboration enhances the understanding of key roles and responsibilities of other professions and leads to improved mutual appreciation [3].

Effective interprofessional collaboration can improve outcomes for patients with advanced cancer and professionals. However, barriers exist in clinical practice. Poor communication between different professionals is one of the greatest barriers, caused for example by professional silos. Other reasons for ineffective communication and collaboration are workforce shortages and high staff turnover in the teams. This results in difficulties in terms of continuity of care and barriers to forming a team identity and developing common mental models and trust [4].

In order to actively promote continuity in communication over the disease trajectory of advanced lung cancer patients in a German thoracic oncology service, an interprofessional patient-centred communication approach (Heidelberg Milestone Communication Approach, MCA [5]) has been developed. This project primarily aimed to improve patient care outcomes (i.e. information needs, quality of life, mood [6]), while strengthening collaboration between specially trained physicians and nurse navigators.

The training within the MCA involved physicians and nurse navigators who worked together in tandem with the patients. They were embedded in a hospital where other healthcare professionals were directly or indirectly affected by the intervention. Although other professions were not an explicit part in the concept, changes in the team work of physicians and nurse navigators may have influenced interprofessional collaboration with other members of the workforce, i.e. therapists who also have a strong relationship with the patients [5]. They met patients with whom therapeutic decisions were made in milestone conversations (MCs). Nurses other than the nurse navigators were in contact with patients at admission, on the ward and during therapy. In addition to the trained resident physicians, physicians in rotation also conduct MCs in tandem with a trained nurse navigator without being trained themselves. Medical assistants have to arrange the schedule. Thus, the MCA not only addresses collaboration between trained physicians and nurse navigators in the tandem, but in a systemic approach also fostered a community of practice [7] among members of the extended healthcare team.

The present mixed-methods study pursues a twofold goal: (1) It aims to present the attitudes to interprofessional collaboration of professions in thoracic oncology before and after the implementation of MCA in a questionnaire survey. (2) To capture unexpected and more complex aspects of implementation, the lived experience of healthcare team members is more deeply explored in qualitative interviews and focus groups.

## Methods

### Setting

The study was conducted at the Department of Thoracic Oncology at the University Hospital Heidelberg, Germany. This hospital is a comprehensive cancer centre with a large catchment area and focuses on thoracic diseases. The Department of Thoracic Oncology provides healthcare for all oncological thoracic diseases, including lung cancer, in oncologic outpatient clinics and 3 oncology and palliative wards.

As part of the novel approach, specially trained physicians and nurse navigators communicate together as a tandem with patients at four defined stages in the illness trajectory in so-called milestone conversations (MCs). Nurse navigators additionally contact patients between clinic appointments to follow-up on questions after appointments, symptoms and well-being. A shared documentation was introduced: Encounters with patients were documented in a shared patient file (color-coded for nurse navigators and physicians) to which other healthcare professionals on the ward also had access.

### Quantitative survey on interprofessional attitudes

#### Study design

To describe interprofessional collaboration of professions in thoracic oncology and detect possible changes in attitudes, staff were asked to complete the validated German translation of the University of the West of England Interprofessional Questionnaire (UWE-IP-D [8]) in a longitudinal study.

Questionnaire data were collected prior to implementation of MCA (t0) in December 2017. MCA was implemented until March 2018. Follow-up data were collected at 4 months directly after the implementation phase (t1), 10 months (t2) and 17 months (t3).

#### Participants

All 120 members of the medical, nursing, administrative, psycho-social, pastoral care, diagnostic and therapeutic professions were included in the survey at the Department of Thoracic Oncology, University Hospital Heidelberg. At each data collection, they received oral and written information including background information on the project, details on participating in the survey and a copy of the questionnaire to fill in.

#### Data collection tools

In the survey, the validated German translation of the University of the West of England Interprofessional Questionnaire (UWE-IP-D [8–10]) was used to assess attitudes to and experiences with interprofessional healthcare. UWE-IP-D is a self-report instrument consisting of 34 items in a set of four scales addressing different themes. Three of the 4 subscales of the UWE-IP-D, i.e. Communication and Teamwork, Interprofessional Interaction, and Interprofessional Relationships, were used. Communication and Teamwork items are measured on a 4-point Likert scale (1 strongly agree, 2 agree, 3 disagree, and 4 strongly disagree) leading to sum scores between 9 and 36, with scores 9–20, 21–25, and 26–36, respectively indicating a positive, neutral, or negative self-assessment of communication and teamwork skills. Interprofessional Interaction and Interprofessional Relationship items are assessed on a 5-point Likert scale (1 strongly agree, 2 agree, 3 undecided, 4 disagree, and 5 strongly disagree). The subscale Interprofessional Interaction takes sum scores between 9 and 45, with scores 9–22, 23–31, and 32–45, respectively, indicating positive, neutral, and negative perceptions of interprofessional interaction. Sum scores on the subscale Interprofessional Relationships vary between 8 and 40, with scores 8–20, 21–27, and 28–40, respectively, indicating positive, neutral, and negative attitudes towards the respondent’s own interprofessional relationships [8]. Additionally, healthcare professionals reported gender and profession (nursing, medical, psycho-social, therapeutic, administrative, or other allied healthcare profession).

#### Data analysis

Descriptive analysis of the quantitative data included calculating subscale sum scores and categorizing each subscale into positive, neutral and negative attitudes [9]. Sum scores are presented as mean with standard deviation overall and across the assessments and categories in absolute and relative frequencies.

### Interviews and focus groups about experiences with the MCA

#### Study design

Qualitative data were collected between November 2018 and April 2019 (12–16 months after implementation of MCA, between t2 and t3 of questionnaire survey). By then, MCA was established and staff informed about the project. For organisational reasons, staff were free to participate in one of the group interviews or to make an appointment for an individual interview. Both group and individual interviews were conducted face-to-face using the semi-structured interview guide in a separate and quiet room on the ward by a health-care researcher with a background in nursing who was not affiliated with the Department of Thoracic Oncology (JB).

#### Participants

For participation in interviews or focus group discussions, a consecutive sample of healthcare staff were invited by research staff who introduced MCA in information rounds on each ward and who were otherwise not involved in the interviews. Only healthcare staff providing clinical care for patients were included (physicians, nurses, therapists). To get an outside view on MCA, nurse navigators as part of the project were excluded. Since all physicians (trained and untrained in MCA) were involved in MCA, they could only provide an inside view. Administrative staff were excluded. All participants gave their written informed consent for study participation.

#### Data collection tools

A semi-structured interview guide addressed the contact to and experiences with MCA (see Additional file 2: Interview guide). The interview guide was developed based on a literature review and in accordance with the objectives of the MCA project. Focus group and Interview questions were oriented towards eliciting open-ended responses to acquire specific information on interprofessional collaboration. The interview guide was pre-tested with one nurse to ensure that all questions were comprehensible. All interviews were digitally recorded, transcribed verbatim and anonymized. The transcripts were compared with the digital recordings to correct any inaccuracies. Data were collected until no additional content could be drawn inductively from the interviews and saturation was reached.

#### Data analysis

Qualitative data were analysed according to Qualitative Content Analysis [11] and to the team-related determinant “Professional Interaction” on the Tailored Implementation in Chronic Diseases (TICD) Checklist [12] as a framework with a focus on organizational aspects to structure collected data into themes and sub-themes. The TICD checklist was adapted to cover the views of untrained professionals (instead of targeted, i.e. trained, professionals only) and the influence on the implementation. Within this approach, a summary of the content was carried out by two female researchers with a background in health services research (M. Sc.) and nursing (JB, SM; not affiliated with the Department of Thoracic Oncology) deleting all expletives and repetitions. Then, the material was coded line-by-line deductively with an a priori developed system of themes derived from the interview guide and the adapted TICD checklist (Communication and influence, Team processes, Referral processes) as well as inductively with additional content emerging from the interviews. All interviews were analysed applying this approach by both data analysts to enhance concordance of coding. The analyses were compared and the coded themes were modified if required. Moreover, all interviews were intensely discussed by the two data analysts in order to ensure agreement. Results were recorded in writing. Credibility was ensured by investigator triangulation throughout the process [13]. Data collection and analysis followed the pre-specified approach lined out in the study protocol [5]. All qualitative data were managed and analysed using MAXQDA 12 (VERBI Software GmbH, Berlin). Quotes presented as examples in this article have been translated from German into English with due diligence and where necessary with slight adaptations to maintain meaning.

### Ethics statement

The study was approved by the Ethics Committee of the University Hospital Heidelberg, Germany (protocol no. S-561/2017). The trial was registered on 22/12/2017 (trial registration no. DRKS00013469).

## Results

### Quantitative survey on interprofessional attitudes

Of all 120 members of the staff, 87 (72,5%; 62 female, 22 male, 3 unknown; 44 nurses, 12 psycho-social staff, 1 diagnostic staff, 4 administration, 7 therapists, 13 physicians, 1 other, 5 unknown) completed the survey at least once (t0: *n* = 20, t1: *n* = 48, t2: *n* = 33, t3: *n* = 25). Only 1 person participated at all data collections, the majority (*n* = 59, 67.8%) participated once making comparisons over time on an individual level impossible.

Attitudes towards communication and teamwork were primarily positive (mean sum score = 17.7, SD = 3.0, min–max: 10–23; positive: *n* = 71, 81.6%, neutral: *n* = 15, 17.2%, negative: *n* = 0, 0%). Attitudes did not differ at among data collections (Table 1).

**Table 1:** Attitudes of staff towards communication and teamwork, interprofessional interaction, and interprofessional relationships

		t0	t1	t2	t3
Communication and Teamwork	n	19	46	32	25
	M (SD)	16.7 (2.3)	17.7 (3.1)	17.4 (3.4)	17.4 (3.4)
	min–max (9–36)	13–20	10–23	11–24	10–24
	positive n (%)	19 (100)	36 (78)	25 (78)	21 (84)
	neutral n (%)	0	10 (22)	7 (22)	4 (16)
	negative n (%)	0	0	0	0
Interprofessional Interaction	n	19	47	33	25
	M (SD)	28.6 (5.6)	28.4 (5.5)	30.5 (4.4)	27.4 (5.5)
	min–max (9–45)	21–45	15–38	22–44	13–38
	positive n (%)	3 (16)	8 (17)	1 (3)	6 (24)
	neutral n (%)	12 (63)	25 (53)	16 (48)	14 (56)
	negative n (%)	4 (21)	14 (30)	16 (48)	5 (20)
Interprofessional Relationships	n	20	47	33	25
	M (SD)	15.6 (4.0)	16.2 (3.7)	15.8 (3.8)	14.8 (3.7)
	min–max (8–40)	8–29	8–26	8–29	8–23
	positive n (%)	19 (95)	42 (89)	31 (94)	24 (96)
	neutral n (%)	0	5 (11)	1 (3)	1 (4)
	negative n (%)	1 (5)	0	1 (3)	0

The majority of the respondents showed neutral attitudes towards interprofessional interaction (mean sum score = 28.5, SD = 5.7, min–max: 13–45; positive: *n* = 14, 16.1%, neutral: *n* = 48, 55.2%, negative: *n* = 22, 25.3%). There were no differences in attitudes across assessments (Table 1).

‘Interprofessional Relationships’ were characterized by primarily positive attitudes overall and across assessments (Table 1; overall mean sum score = 16.0, SD = 3.6, min–max: 8–29; positive: *n* = 80, 92.0%, neutral: *n* = 6, 6.9%, negative: *n* = 1, 1.1%).

### Interviews and focus groups about experiences with the MCA

Qualitative interviews with 15 staff (3 physicians, 11 nurses, 1 therapist) were conducted, which included 4 individual interviews (physicians, therapist) and 5 group interviews with 2–3 participants (nurses) each. Saturation was reached after 2 individual and 4 group interviews. The mean duration of the qualitative surveys was 26 min (range 9 min to 38 min). Regarding factors associated with implementation, the participants addressed the TICD interdependent main themes regarding interprofessional collaboration: (1) Communication and influence, (2) Team processes, and (3) Referral processes.

#### Theme 1: communication and influence

The theme “Communication and influence” describes the extent to which the support of the intervention is influenced by professional opinions and communication (adapted from [12]). Related to the MCA, this theme comprises “Knowledge about the MCA”, “Role of patients”, and “Own role in team”.

##### Knowledge about the MCA

Each of the staff interviewed had already heard of the MCA. Their knowledge in detail depended on the contact they had with the project and its participants: physicians were more informed by being part of the project, others knew it from a distance without being directly involved.

##### Role of patients

Patients acted as deliverers of information between the MCA team and other staff.

The majority of interviewees working on the ward mentioned that they could not identify the patients participating in the MCA. While some staff received information from their patients about their participation in the MCA, other patients never mentioned it. Patients who mentioned the MCA to the interviewees reported positive experiences and an enhancement of the treatment process.

"I have heard from patients that the contact to the MCA team is appreciated and maintained […]. And I always find that in itself […] an enrichment.” (interview 2, physician).

##### Own role in team

Even if the interviewed physicians had not received the MCA training, they were able to report how the intervention influenced the roles they had in the team. Physicians for whom MCA was a new experience sometimes felt overruled in their therapeutic decisions when working together with a (trained) nurse navigator.

“I remember a situation with a patient who did not feel well, and then the nurse had already talked to her about discontinuing therapy. That was, for me in that moment it was a bit outside their scope.” (interview 3, physician).

In addition, therapeutic conversations conducted together with a patient were perceived as physician-dominated with the nurse navigator in a secondary role.

Tasks and roles of the different team members were not explicitly defined in the project. Team members could flexibly adapt their roles and responsibilities as part of the implementation process. Over time, clearer definitions of tasks and roles emerged and led to a higher degree of acceptance of shifted responsibilities.

“That was at the beginning when the role of the MCA team wasn’t exactly clear, that was just perhaps a little hyperactivity. Otherwise there is not much to criticise.” (interview 3, physician).

“There were conversations which were physician-centred and the nurses had a passive role, but they contacted the patient afterwards. So, there was less direct participation during the conversations” (interview 2, physician).

Nurses who were not part of the physician-nurse tandem still perceived evolution in the doctor-nurse relationship distinct from MCA. From their point of view, the therapeutic conversations generally were conducted in partnership and not physician-dominated.

"There are the somewhat older physicians who are perhaps used to talking and the nurse listening, and today it is already the case with many of them that the conversation is held together. The physician and the nurse" (focus group 1, nurse 2).

#### Theme 2: team processes

The theme “Team processes” describes the extent to which teams are involved to support implementation (adapted from [12]). Related to the MCA, this theme comprises Team competencies, Imparting information and Barriers.

##### Team competencies

Team competencies include aspects and areas of competence that belong to interprofessional collaboration associated with the MCA. Therefore, only staff who were actually in direct contact to the MCA could reflect their experiences first-hand. In our sample, this concerned physicians only. Due to rotation within the hospital, not all of them were trained in MCA but sometimes conducted therapeutic patient conversations in a tandem with a trained nurse navigator.

With the introduction of the MCA, perception of physicians and nurse navigators in the tandem differed about how and when to deliver information to the patients, especially about the prognosis. From the physician’s point of view, nurse navigators wanted the patients to be fully informed from the beginning, while physicians provided information in small pieces for each patient individually. Over time, the tandems found a way to lead satisfactory conversations.

Members of a tandem perceived each other as supportive in providing information, preparing patients for the conversations, conducting and debriefing the conversations, and documentation. The collaboration within the interprofessional tandem was evaluated positively. Feedback after the MCs was appreciated. Other team members were also perceived as providing emotional support.

“Especially in critical situations, you don’t have to carry the load of telling of progression or saying you cannot do any more (tumor centered therapy), you don’t have to carry that alone. That should not be underestimated. So, I think this is also a moral support for the physicians, if there is someone else and supporting the people.” (interview 3, physician).

Collaboration within the team led to more knowledge about the patient: the team regularly exchanged information about the situation and clinical status of the patient. Ambiguities were timely clarified. Physicians became more aware of the support provided by nurse navigators and used it more often. For patients who were perceived as needing more support, the nurse navigator was informed and able to schedule additional time for debriefing with the patients. MCA provided an agenda for topics to be addressed in a conversation. Although structured, the conversations sometimes took unintended turns and therefore lasted longer than planned. Still, it was considered an advantage when nurse navigators addressed patient-relevant issues during the conversation, debriefed with the patients and answered questions arising after the scheduled conversation. From the physicians' point of view, the nurse navigators tended to see the support needs of the patients during the MCs. Nurse navigators were an additional support, which helped to interpret the patient's statements and to address important issues again at a later time.

“During the conversation, there is always one or another issue, that the nurses notice and address. I think that’s great […] there are just questions they then can clarify outside (after the conversation). I consider this very good for patients because we are aware that patients receive a lot of information […] and of course coordinating appointments keeping deadlines, medication, that is very complicated and for them (patients) very supportive. I also had challenges at the beginning but now it works well and both physicians and patients benefit very much.” (interview 4, physician).

Although the participating staff perceived advantages in bringing different professional perspectives together and acknowledged the problem-solving opportunities this offered, staff not involved in the tandem highlighted difficulties they experienced figuring out how to include MCA into their daily routine.

“I can imagine that it (the MCA) has advantages, because everybody is sharing their view on the patient, and how problems can be solved, for the patient but also within the team maybe.” (interview 2, physician).

"Well, there are some of our colleagues who cannot yet integrate MCA into our daily activities. […] Therefore I believe that MCA is a real support for the patient, but it is not yet a benefit for us." (focus group 1, nurse 1).

Some interviewees reported no change in interprofessional teamwork from the introduction of the MCA. But they stressed the point of having had a good collaboration beforehand.

“Here it (interprofessional teamwork) is very good anyway. Otherwise it wouldn’t work. It is trusting and very good. Still. I would be lying if I’d say this has all become much better. It is just good.” (interview 3, physician).

##### Imparting information

Means by which information was passed to other members of the staff, had room for improvement in the transparency of the MCA. From the interviews, it emerged that staff other than the nurse navigators and physicians who were trained and/or conducted milestone conversations (MCs) were not informed about details of the MCA project. Nevertheless, the project and the exchange with the MCA team provided additional (unstructured) information to the wards.

"It happens from time to time that an MCA-nurse comes and says: ‘(Name of the patient) is not well. I have already called them once, they come (here), they have this and that.’ That's information we usually don't get." (Individual interview 3, physician).

To strengthen information exchange, interviewees articulated the wish to receive a summary of the MCs to improve patient support. As part of the project, the nursing staff on the ward had access to all written information on MCs. Still, they expressed the wish to have a brief oral explanation of what was addressed in the MCs and what the patient's needs were. With this information, they could continue and improve the patient's care.

"A brief feedback session [would be good] if they are coming to the ward to see some patients anyway." (focus group 2, nurse 3).

“Everything is documented. That’s new, that it is in green now. And on top, it says in big letters ‘MCA patient’, so you know,’I can call someone if there is something where I cannot answer or don’t have time’.” (focus group 5, nurse).

##### Barriers

Changes in interprofessional collaboration initiated by the MCA can be best understood by looking at logistic influences on and by the MCA. At the beginning of the implementation, some of the MCs did not proceed as planned. Both organizational and interpersonal factors were identified as reasons for this. In particular, the lack of a fixed place for follow-up calls or debriefing with the patient after an MC was mentioned in the area of organizational aspects.

In terms of interpersonal aspects, a barrier was seen at the beginning of the implementation process in that the distribution of roles and the interaction within the tandem was not yet defined. A lack of clarity regarding roles and interaction led to some insecurity on the part of physicians and nurses, which can affect the quality of the counseling. The exchange of information was not only dependent on the individual, but also on surrounding conditions, such as time. Some staff indicated that a good exchange about the patient should take place during the afternoon shift, as many time-consuming nursing and therapeutic measures were carried out on the morning shift.

#### Theme 3: referral processes

The theme “Referral processes” describes processes of transferring patients within and between outpatient departments and inpatient wards and interprofessional communication (adapted from [12]). Within the MCA, this theme focused on Cross-sectoral communication.

##### Cross-sectoral communication

Cross-sectoral communication refers to the communication between the outpatient department and the inpatient wards. Overall, staff stated that effective communication between the wards and the outpatient department played an important role for them, as it enabled them to optimize patient care and to provide it in a targeted manner. Nevertheless, communication in the context of the MCA was perceived as controversial. The exchange about patients depended on the relationship between individual members of the staff. Advantages were particularly observed when the nurse navigator was both part of the interprofessional tandem and additionally worked part time on the ward. Nurse navigators in the MCA who had good relationships with staff on the ward communicated more about patients' individual situations.

"I can say that it is sometimes depending on the person, the nurse who has worked in our ward, for example, I am more in contact with her because I interact with her directly […].” (focus group 1, nurse 2).

One interviewee stated that the tight schedule on the wards allowed only narrow time frames for exchanging information, which often affected communication. The interviewee also described their reluctance to talk to someone if they felt that the other person did not have the time.

"There's bustle, pressure on from care requirements, I'd say, especially on the ward [name of the ward], so that I'm already very reluctant to approach the doctors individually.” (interview 1, therapist).

## Discussion

This study explored attitudes towards interprofessional collaboration of professions in thoracic oncology and factors for implementation of the MCA. Participants in the survey, who were mainly not involved in the MCA, rated their communication and teamwork skills high and perceived their relationship with healthcare colleagues positively. In the interviews it became apparent that quality of interprofessional collaboration was related to time and personal relationships. Implementation required adjustment and adaptation over time which included feeling insecure about responsibilities and role assignment [14]. Physicians and nurse navigators supported each other during the MCs, and the exchange with the wards is also perceived as functioning, especially if a nurse navigator has a good connection to the ward staff. From the wards, an increased transparency regarding the project is desired in order to strengthen the collaboration and the knowledge about the MCA.

Results from interviews and focus group discussions reflected in the attitudes to and experiences with interprofessional work among staff in the oncology department assessed in the questionnaire survey. Participants in the questionnaire survey appreciated communication and teamwork as well as interprofessional relationships. The assessment of interprofessional interaction was primarily in the neutral range indicating room for improvement. A strong hierarchy among healthcare professionals and widespread stereotypes [15] is not conducive to effective interprofessional interactions. To achieve successful interprofessional interactions, a variety of aspects have to be considered, including equal and collaborative relationships between different professional groups, (un)biased views, open communication, respect and the degradation of hierarchical status [10]. Some of these issues are more difficult to address within interprofessional communication training than others. While openness to communication is a prerequisite for applying training into practice, traditionally hold views on status are less easily overcome [16]. The fact is that this barrier exists in practice and has an influence on an individual's own role within the team which was shown in the interviews. During the implementation of the MCA, physicians and nurses initially struggled to define their roles and responsibilities, but managed to adapt to the challenges of the approach and to strengthen interprofessional interaction over time. For staff members not directly involved in the MCA, it was more difficult to adapt their roles and tasks in relation to the MCA. Future implementation of interprofessional concepts thus need to take into account not only attitudes of individuals but also existing team structures to successfully foster interprofessional interactions.

Further aspects of effective collaboration represent successful communication and teamwork. Communication and teamwork involve exchanging opinions, explaining issues and feeling comfortable working in a group [10]. They are core features of the MCA [17]. For a successful implementation of these aspects, physicians and nurses who put the intervention into practice need to be motivated to adhere to the concept goals and consider themselves an essential part of the implementation [12]. The positive attitude towards communication and teamwork was highlighted in the results of the questionnaire survey and was partly mirrored in the interviews. Nevertheless, interviews showed that physicians in particular did not consider themselves part of the MCA team and referred to "them" whenever current MCA issues were discussed, even when they were involved in MCA discussions with patients. Possible reasons for this are seen in the fact that the "team" did not seem to be defined. Next to the tandem with alternating nurse-physician duos, nurse navigators defined themselves as a team. Beyond the professionals directly involved, the importance of transparency about the concept was underestimated at the beginning of the project, thus hindering the support by the extended healthcare team.

On the other hand, positive experiences with the MCA supported maintenance and further facilitation of already implemented aspects. Even if participants in the interviews did not receive formal training but were involved in patient conversations, they observed alleviation through the MCA both for themselves and the patients. The participating healthcare professionals not directly involved in the MCA received additional information about the program from the patients. Positive experiences were reported both by patients and healthcare professionals. Transparency about the approach and involving healthcare professionals not directly conducting patient consultations to keep the whole team informed, foster a community of practice [7] and indirectly strengthen care by giving the patients a generated feeling of team-delivered patient-centred care [18].

Improving patient-centred care is a common goal of interprofessional practice which involves high quality teamwork [19]. Communication is a prerequisite for teamwork. Especially in interprofessionally conducted conversations with patients, participants need to know their own part and the role of the team members. Tasks and responsibilities have to be prepared and coordinated beforehand to achieve successful collaboration with shared accountability [20] without denying individual characteristics of healthcare staff and the team competencies emerging from their interpersonal relationships [21].

Apart from the task of performing trajectory-based conversations in a nurse-physician tandem, prerequisites like sufficient time and space for conducting conversations with the patients as planned posed an additional challenge. Those context factors (“Availability of necessary resource” in the TICD checklist [12]) were not in the focus of this study but emerged as important influences on both fidelity of the intervention and perception of the teamwork in the interviews. Future implementations should pay stronger attention to context factors (e. g. by using a coding model [22] based on the Consolidated Framework for Implementation Research [23]). Identification of logistic barriers and taking up measures to lower them will not only foster the implementation but also raise the motivation of staff not directly involved in the project, thus strengthening the team and interprofessional interaction [24].

### Limitations

Although this mixed-methods study provided a plethora of results, some limitations have to be considered. First, this study was conducted at one site only, since MCA was developed and implemented there as a pilot. The observations and reported experiences might be highly depended on the individual clinic. Some results are generalizable and could apply to other hospital settings, i.e. the change of roles in a team with the introduction of new responsibilities which may lead to long-term changes in interprofessional attitudes and skills. We could not draw conclusions about a change in attitude in our quantitative survey, since only few participants provided data at more than one data collection. On the other hand, the experiences reported in the interviews and focus groups suggest a shift in the perception of responsibilities and interprofessional collaboration.

Interviews and focus groups were conducted with staff who self-selected for participation and who were interested in the topic. Their opinions might not reflect the impressions of the overall healthcare staff. Especially the views of allied health professionals could not be considered. Although every staff member could participate, their not taking part might allude to too little information of the program and to not perceiving any change. Additionally, group interviews might inhibit individual participants from speaking frankly. Our group discussions were characterized by a high level of trust; critical comments were expressed freely.

In our study, a change in interprofessional collaboration in the whole department after introducing an interprofessional approach to a small number of healthcare professionals could not be shown during the course of the study. To establish a community of practice in an interprofessional team requires more time for adaptation processes and changes of attitude. Still, our participants reported experiencing advantages of the MCA which further consolidation might be built on.

## Conclusions

In summary, our study showed both positive experiences and potential shortfalls in the implementation of the MCA. Aspects were identified which need a stronger focus in future introduction of trajectory-based conversations in an interprofessional tandem, i.e. preparation for conversations within the tandem and information exchange beyond the tandem to allow patient-centred care by an interprofessional team involving physicians, nurses, therapists, psychosocial professionals and others. Changing roles and responsibilities in the team may initiate a paradigm shift throughout the organisation.

## Supplementary Information

**Additional file 1. **Codes MCA staff.

**Additional file 2.** Interview guide.

## Data Availability

The datasets generated and analysed during the current study are not publicly available since participants did not consent to publication. However, anonymized and/or aggregated data are available from the corresponding author on reasonable request.

## Abbreviations

MCA: Milestone Communication Approach
MCs: Milestone Conversations
SD: Standard deviation
TICD: Tailored Intervention in Chronic Diseases
UWE-IP-D: German version of the University of the West of England Interprofessional Questionnaire
