# Supplementary material for: Factors related to implementation of an interprofessional communication concept in thoracic oncology: a mixed-methods study
Source: BMC Palliat Care. 2022 May 26;21:89. doi: 10.1186/s12904-022-00977-6 (PMC9134656; doi:10.1186/s12904-022-00977-6)
Supplement: Supplementary file 1 — Additional file 1. Codes MCA staff. [file 12904_2022_977_MOESM1_ESM.docx]

Codes MCA staff

| Topic | Code | Definition |
| --- | --- | --- |
| Communication and influence | Knowledge about MCA | Level of awareness of the MCA |
|  | Patient perspective 🡪 Role of patients | Patients inform team members about MCs |
|  | Closeness and distance 🡪 own role in team | Membership of team members in one or more teams and interaction taking place within them |
| Team processes | Influence of the project 🡪 team competencies | Interprofessional component associated with MCA |
|  | Exchange about patient 🡪 imparting information | Communication about the patient in the multiprofessional team |
|  | barriers | Shortcomings affecting implementation |
|  | Solution approaches 🡪 barriers | Solutions for shortcomings |
| Referral processes | Cross-sectoral communication | Perception of communication between outpatient department and wards |
