# Supplementary material for: Factors related to implementation of an interprofessional communication concept in thoracic oncology: a mixed-methods study
Source: BMC Palliat Care. 2022 May 26;21:89. doi: 10.1186/s12904-022-00977-6 (PMC9134656; doi:10.1186/s12904-022-00977-6)
Supplement: Supplementary file 2 — Additional file 2. Interview guide. [file 12904_2022_977_MOESM2_ESM.docx]

Topics in interviews and focus groups about MCA with staff

| Topic | Questions (suggested) |
| --- | --- |
| Introduction of interview partners (name, affiliation, function) | Would you please introduce yourself, where you are working and what your function is? |
| short summary of MCA by interviewer |  |
| If desired: Task arranging paper shapes to visualize own position in team (including MCA team) and department | Here are some paper shapes depicting physicians, nurses, therapists, the different wards, the MCA team. Would you pick a shape representing yourself and arrange the others as how closely you and they work together from your point of view? |
| Experiences with MCA | What are your experiences with the MCA (team)? |
| Reflection about own position and collaboration | Where do you see yourself with respect to MCA? |
| Perceived influence of MCA on collaboration | In your opinion, how does MCA influence collaboration? |
| Perceived advantages of MCA | In your opinion, what are the advantages of MCA? |
| Perceived barriers for implementation of MCA and how to address them | You perceived (getting back to experiences reported) as difficult. How could it be made better? |
| Summary of most important aspects |  |
